# C3 mutation-associated atypical hemolytic uremic syndrome with severe renal dysfunction and hypertensive emergency successfully treated with ravulizumab and sacubitril/valsartan: a case report

**DOI:** 10.1186/s12882-026-04797-1

**Published:** 2026-02-12

**Authors:** Hiroki Yanagidani, Yujiro Maeoka, Maria Yoshida, Mayuko Ueda, Mari Kumano, Akira Takahashi, Aiko Okubo, Noritoshi Kato, Shoichi Maruyama, Takao Masaki

**Affiliations:** 1 Department of Nephrology, Hiroshima University Hospital, 1-2-3 Kasumi, Minami-ku, Hiroshima, 734-8551 Japan; 2 Department of Nephrology, Nagoya University Graduate School of Medicine, 65 Tsuruma-cho, Showa-ku, Nagoya, Aichi Japan

**Keywords:** Atypical hemolytic uremic syndrome (aHUS), C3 mutation, Ravulizumab, Angiotensin receptor–neprilysin inhibitor (ARNI), Hypertensive emergency (HE), Thrombotic microangiopathy (TMA), Renal recovery

## Abstract

**Background:**

Atypical hemolytic uremic syndrome (aHUS) is a rare complement-mediated thrombotic microangiopathy characterized by microangiopathic hemolytic anemia, thrombocytopenia, and acute kidney injury. Although C5 inhibitors, such as eculizumab and ravulizumab, have markedly improved outcomes, renal recovery remains limited in cases complicated by hypertensive emergency (HE) or malignant hypertension, likely due to complement-independent vascular injury. Angiotensin receptor–neprilysin inhibitor therapy exerts dual actions of renin–angiotensin system blockade and augmentation of natriuretic peptide signaling. Additionally, this therapy has recently demonstrated renal benefits in thrombotic microangiopathy with HE compared with conventional renin–angiotensin system inhibitors. However, the combined use of an angiotensin receptor–neprilysin inhibitor with a C5 inhibitor in aHUS with HE has not been reported.

**Case presentation:**

A 57-year-old Japanese man presented with marked anemia, severe renal dysfunction (serum creatinine concentration: 10.19 mg/dL; eGFR 5 mL/min/1.73 m^2^), and hypertensive emergency (blood pressure: 198/102 mmHg). Laboratory examinations showed hemolytic anemia and thrombocytopenia, leading to a diagnosis of thrombotic microangiopathy. Plasma exchange was performed, and intravenous antihypertensive therapy was initiated on day 1. Ravulizumab was started on day 2 after thrombotic thrombocytopenic purpura and Shiga toxin-associated HUS were excluded, and aHUS was clinically suspected on the basis of isolated C3 hypocomplementemia. Despite the requirement for initial hemodialysis, renal function gradually improved, allowing withdrawal of dialysis by day 14. A renal biopsy showed thrombotic microangiopathy with intravascular thrombi and concentric arteriolar wall thickening resembling onion-skin lesions. Consequently, sacubitril/valsartan was initiated on day 23, resulting in further renal recovery (creatinine concentration: 3.79 mg/dL on day 22 to 1.23 mg/dL at 1 year; eGFR 14 to 48 mL/min/1.73 m^2^ at 1 year) accompanied by improved blood pressure control. Genetic testing identified a heterozygous C3 c.493G > T (p.Val165Phe) variant, which confirmed C3 mutation-associated aHUS.

**Conclusions:**

We report the first case of C3 mutation-associated aHUS with HE, which was successfully treated with a combination of ravulizumab and sacubitril/valsartan. The renal improvement in this patient may suggest a potential, but unproven, contribution of angiotensin receptor–neprilysin inhibitor-based renin–angiotensin modulation to kidney recovery when added to complement inhibition in aHUS complicated by HE, although its independent effect remains unclear. Further cohort studies are warranted to clarify its potential therapeutic synergy.

## Background

Atypical hemolytic uremic syndrome (aHUS), Shiga toxin-producing *Escherichia coli*-associated HUS, and thrombotic thrombocytopenic purpura are collectively referred to as the major thrombotic microangiopathies (TMAs). TMA is a multisystemic condition defined by a triad of acute renal failure, microangiopathic hemolytic anemia, and thrombocytopenia [1]. In the kidney, characteristic findings of TMA include glomerular intracapillary and/or arteriolar thrombosis, fragmented red blood cells within capillary lumens, and focal ischemia [2, 3].

aHUS is a prototypical disorder caused by overactivation of the alternative complement pathway. This syndrome is associated with mutations in complement regulators, such as complement factor H, complement factor I, and membrane cofactor protein (CD46). This syndrome is also associated with activating components, such as complement component 3 (C3) or complement factor B of the C3 convertase, which is the central enzymatic complex of the alternative complement pathway [4]. Eculizumab, a humanized monoclonal antibody that shows terminal complement activation by blocking cleavage of complement component 5 (C5), was approved for treating aHUS in 2011 and revolutionized its clinical management [5]. Ravulizumab, a new long-acting C5 inhibitor, introduces four amino acid substitutions in the complement-binding and neonatal Fc regions of eculizumab, enabling efficient recycling and augmented endosomal dissociation of C5 [6]. Ravulizumab has shown similar efficacy to eculizumab in improving TMA [7] and was approved for aHUS in 2019. Although these C5 inhibitors improve the overall prognosis of aHUS, recent study suggests that renal survival may not significantly improve with eculizumab in patients with hypertensive emergency (HE) [8]. HE is defined as systolic blood pressure > 180 mmHg or diastolic blood pressure > 120 mmHg accompanied by end-organ damage, which itself can precipitate TMA [8]. HE is a well-recognized and common manifestation of aHUS, affecting approximately half of patients [9]. Therefore, aHUS with HE appears to involve a complex pathophysiology, and is characterized not only by dysregulation of the complement system but also by superimposed complement-independent vascular injury.

The angiotensin receptor–neprilysin inhibitor (ARNI) sacubitril/valsartan belongs to a novel class of drugs, which combines inhibition of endogenous natriuretic peptide degradation with blockade of renin–angiotensin system (RAS) activation [10, 11]. There is mounting evidence from large, randomized, clinical trials that ARNIs are superior to angiotensin-converting enzyme (ACE) inhibitors/angiotensin receptor blockers (ARBs) in providing cardiovascular and renal benefits in patients with heart failure and chronic kidney disease [12, 13]. Compared with ACEs/ARBs, an ARNI has been shown to improve kidney recovery in patients with malignant hypertension/HE [14], which is a condition in which the renin–angiotensin–aldosterone system is highly activated [15, 16]. Therefore, effective blood pressure control and RAS blockade are crucial. Although severe hypertension and arteriolar wall thickening with an onion skin appearance and luminal occlusion, which are characteristic features of malignant hypertension/HE, are frequently observed in patients with aHUS [17], there have been no reported cases of aHUS treated with a combination of C5 inhibition and an ARNI. Additionally, only a single case report has described complement factor H mutation-associated aHUS with HE treated with ravulizumab [18].

We report a case of C3 mutation-associated aHUS with HE and severe renal dysfunction, which required temporary dialysis, but was successfully treated with ravulizumab and an ARNI, leading to a decrease in serum creatinine concentrations from 10.19 mg/dL to 1.23 mg/dL.

## Case presentation

A 57-year-old Japanese man with no notable medical history visited a local clinic with gastric discomfort and was referred to a secondary hospital because of marked anemia (hemoglobin: 6 g/dL) and severe renal dysfunction (serum creatinine [Cr]: 6.0 mg/dL). He was admitted the same day with suspected TMA owing to the presence of schistocytes and thrombocytopenia, and was transferred to our institution several days later for further evaluation and treatment. A routine health check several weeks earlier had shown mild anemia (hemoglobin: 13 g/dL) and mild renal impairment (Cr: 1.3 mg/dL), which suggested that both conditions had already begun to progress at that time. Approximately 2 months previously, all members of his household had been diagnosed with coronavirus disease 2019 (his parents tested positive via antigen testing and his eldest son was positive via a home test kit), although he was not tested despite experiencing mild upper respiratory symptoms. He was a current smoker (up to 15 cigarettes each day) and was not taking any medications.

Upon admission, the patient’s blood pressure was 198/102 mmHg and heart rate was 65 beats/minute. On physical examination, the palpebral conjunctiva showed mild pallor, and no skin rash was observed. Breath sounds were clear. No peripheral edema or meningeal signs were noted. The patient was alert with a Glasgow Coma Scale score of 15, hemodynamically stable, and showed no signs of respiratory distress or focal neurological deficits, indicating that his general condition was relatively preserved. Laboratory testing showed hemolytic anemia (elevated lactate dehydrogenase concentration and low haptoglobin concentration, and schistocytes), thrombocytopenia, and renal failure with proteinuria and hematuria (Cr: 10.19 mg/dL, urine protein-to-creatinine ratio: 3.27 g/gCr, red blood cells: 30–49/high-power field). The serum cortisol concentration, plasma renin activity, aldosterone concentration, and catecholamine concentration were within the normal range. Autoimmune serologies, including antinuclear antibody, were negative (Table 1). Abdominal ultrasonography showed no renal atrophy or hydronephrosis (right kidney: 9.6 × 5.7 cm, left kidney: 9.8 × 4.4 cm). Non-contrast head computed tomography showed no evidence of hypertensive encephalopathy. Non-contrast chest-abdominal computed tomography showed no pulmonary nodules or bowel wall thickening. Fundoscopic examination revealed hypertensive retinopathy with cotton-wool spots and retinal hemorrhages. Transthoracic echocardiography demonstrated preserved left ventricular systolic function, with an ejection fraction of 58%, and no significant wall motion abnormalities. Based on the triad of hemolytic anemia, thrombocytopenia, and acute kidney injury, a diagnosis of TMA was made.

**Table 1:** Summary of the patient’s laboratory results

Parameter	Value	(normal range)
*Urine*		
Urine specific gravity	1.02	
pH	5.5	
Urine protein/creatinine ratio (g/gCr)	3.27	(<0.15)
Red blood cells (/HPF)	30–49	(<5)
β2 MG (mg/L)	0.84	(0.03–0.37)
NAG (IU/L)	50.2	(0–11.5)
*Blood*		
White blood cells (/µL)	8560	(3040–8540)
Red blood cells (10^4^/L)	256	(378–499)
Hemoglobin (g/dL)	8.5	(10.8–14.9)
Hematocrit (%)	24.9	(35.6–45.4)
Platelets (10^4^/L)	11.5	(15–36)
T-Bil (mg/dL)	1.6	(0.4–1.5)
D-Bil (mg/dL)	0.3	(0.1–0.3)
AST (U/L)	41	(13–33)
ALT (U/L)	25	(8–42)
LD (U/L)	1119	(124–222)
Total protein (g/dL)	5.1	(6.7–8.3)
Serum albumin (g/dL)	3.1	(4–5)
Blood urea nitrogen (mg/dL)	74.7	(8–20)
Creatinine (mg/dL)	10.19	(0.4–0.7)
eGFR (mL/min/1.73 m^2^)	5	(>90)
Sodium (mmol/L)	137	(138–146)
Potassium (mmol/L)	5.1	(3.6–4.9)
Chloride (mmol/L)	106	(99–109)
Calcium (mg/dL)	7.5	(8.6–10.4)
Phosphorus (mg/dL)	5	(2.5–4.7)
Uric acid (mg/dL)	10.1	(2.3–7)
Plasma glucose (mg/dL)	102	(70–109)
Hemoglobin A1c (NGSP) (%)	4.1	(4.6–6.2)
C-reactive protein (mg/dL)	0.02	(<0.2)
PT-INR	1.02	
APTT (s)	28.1	(24–34)
Fib (mg/dL)	299.8	(200–400)
Immunoglobulin G (mg/dL)	648	(870–1700)
Immunoglobulin A (mg/dL)	116	(110–410)
Immunoglobulin M (mg/dL)	46	(46–260)
CH50 (U/mL)	32.8	(30–46)
C3 (mg/dL)	38	(86–160)
C4 (mg/dL)	28	(17–45)
Haptoglobin (mg/dL)	1	(19–170)
Anti-nuclear antigen	Negative	Negative
Anti-neutrophilic cytoplasmic antibody	Negative	Negative
Anti-glomerular basement membrane antibody	Negative	Negative
HBs-Ag	Negative	Negative
HCV-Ab	Negative	Negative
Renin (ng/mL/H)	1.5	(0.2–2.3)
Aldosterone (ng/dL)	7.1	(0.4–8.21)

Cr: creatinine, HPF: high-power field, β2MG: β2-microglobulin, NAG: N-acetyl-β-d-glucosaminidase, T-Bil: total bilirubin, D-Bil: direct bilirubin, AST: aspartate transaminase, ALT: alanine transaminase, LD: lactate dehydrogenase, eGFR: estimated glomerular filtration rate, NGSP: National Glycohemoglobin Standardization Program, PT-INR: prothrombin time-international normalized ratio, APTT: activated partial thromboplastin time, Fib: fibrinogen, CH50: 50% hemolytic complement activity, C3: complement component 3, C4: complement component 4, HBs-Ag: hepatitis B virus antigen, HCV-Ab: hepatitis C virus antibody

**Fig. 1:**
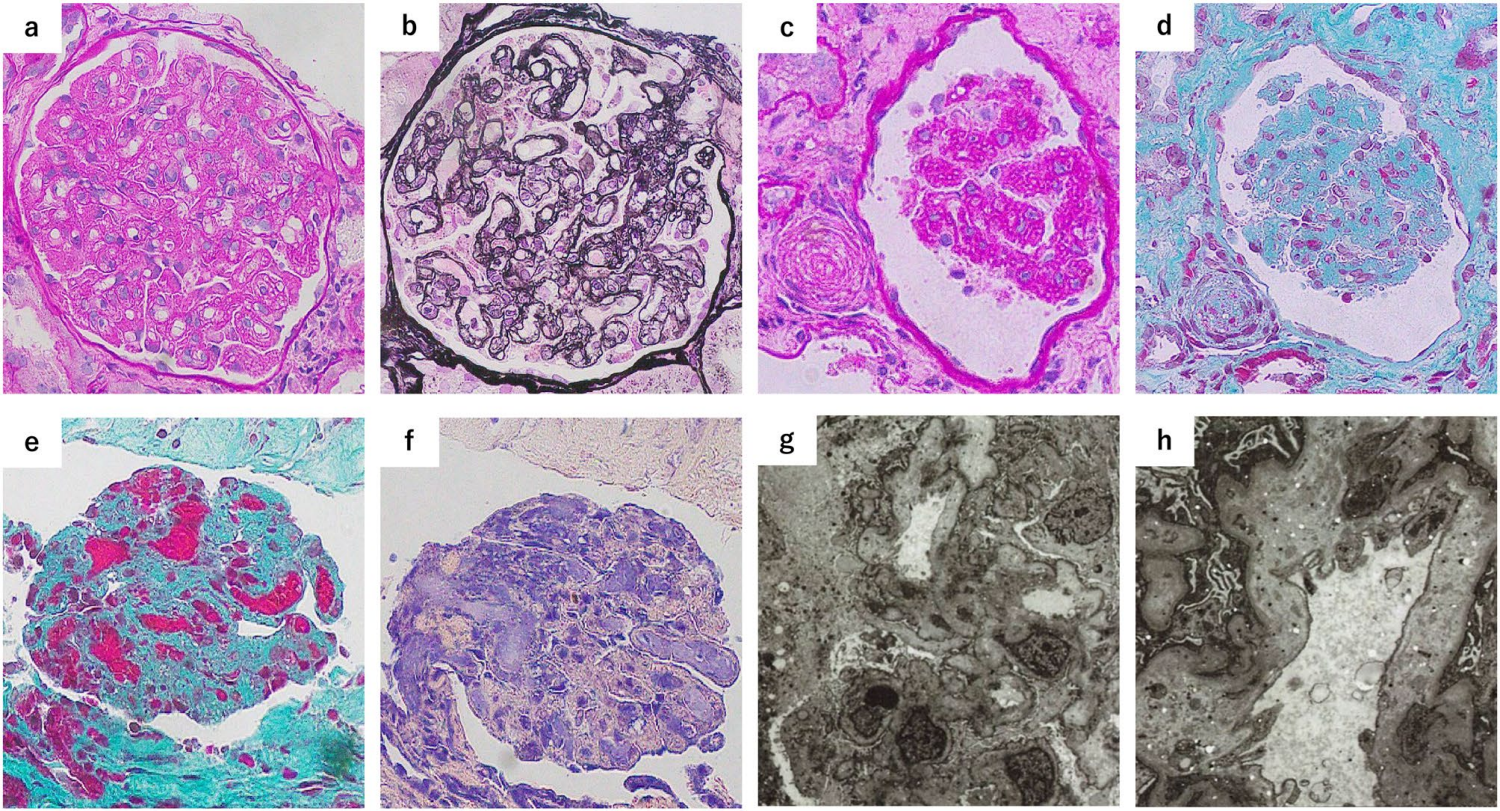
Light microscopic images of the renal biopsy. (**a**–**d**) Periodic acid–Schiff staining (**a**: ×400) and periodic acid–methenamine–silver staining (**b**: ×400) show double contours of the glomerular basement membrane. Periodic acid–Schiff (**c**: ×400) and Masson’s trichrome staining (**d**: ×400) show an onion skin lesion in arterioles. (**e, f**) Masson’s trichrome staining (**e**: ×400) and phosphotungstic acid hematoxylin staining (**f**: ×400) show thrombi within the glomerular capillaries. (**g, h**) Electron microscopy shows subendothelial lucent expansion observed globally (**g**: ×1500, **h**: ×5000)

**Fig. 2:**
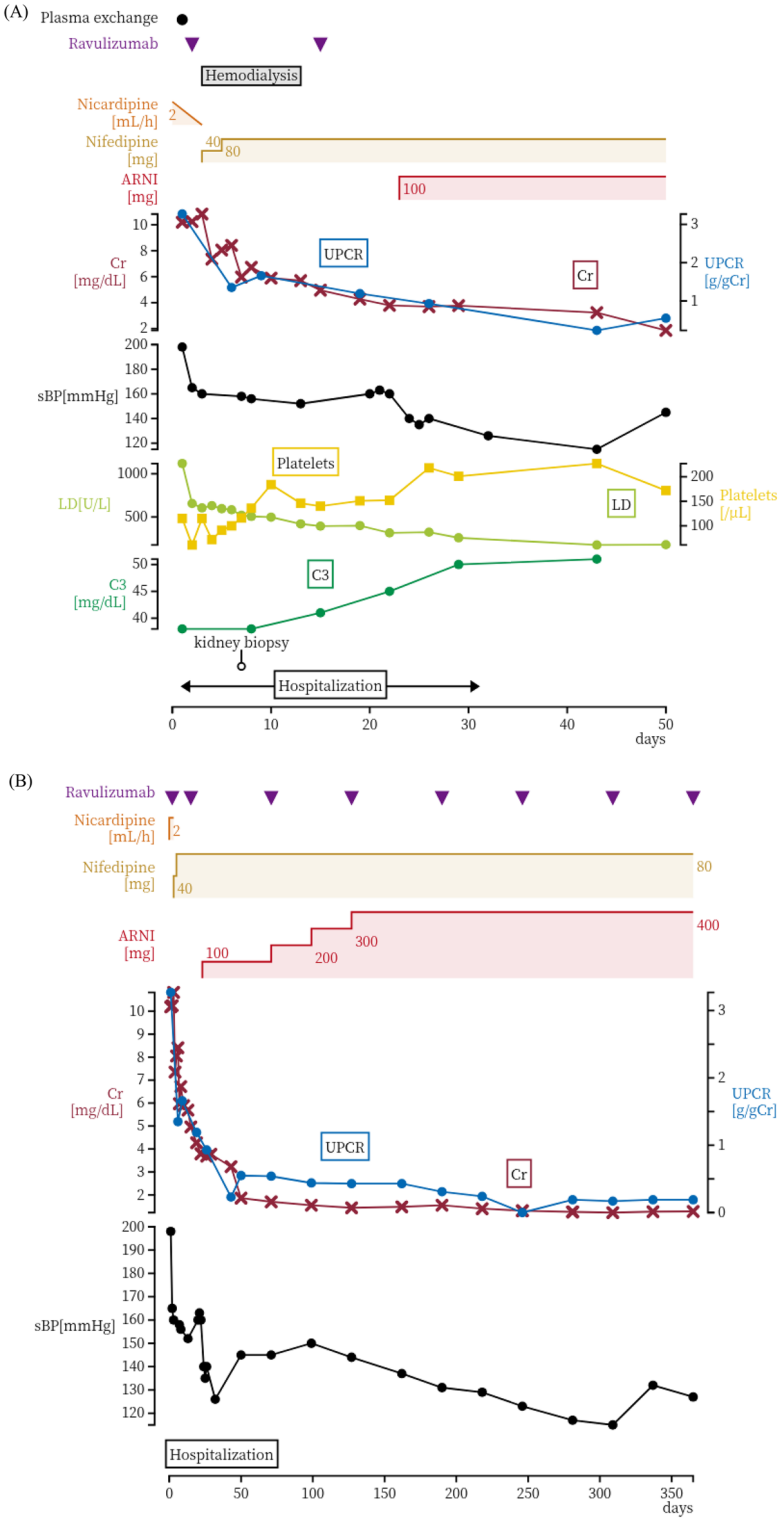
Clinical course of the patient. (**A**) Acute-phase clinical course during hospitalization. (**B**) Long-term clinical course up to 1 year after disease onset. Changes in serum Cr (brown lines), the UPCR (blue lines), sBP (black lines), LD (yellow green lines), platelets (yellow lines), and C3 (green lines). ARNI: angiotensin receptor–neprilysin inhibitor, UPCR: urine protein/creatinine ratio, Cr: creatinine, sBP: systolic blood pressure, LD: lactate dehydrogenase, C3: complement component 3

On day 1, intravenous antihypertensive therapy with nicardipine was initiated, and plasma exchange was performed because thrombotic thrombocytopenic purpura could not be excluded. On day 2, ADAMTS13 activity was found to be within the normal range (38%), effectively ruling out thrombotic thrombocytopenic purpura. The absence of gastrointestinal symptoms, such as diarrhea or hematochezia, and a negative stool culture, ruled out Shiga toxin-producing *Escherichia coli*-associated hemolytic uremic syndrome. With no evidence of an underlying disease and isolated C3 hypocomplementemia (38 mg/dL), with a normal C4 concentration (28 mg/dL) (Table 1), activation of an alternative complement pathway was suspected, leading to a clinical diagnosis of aHUS. Therefore, the anti-C5 monoclonal antibody ravulizumab was administered on days 2 and 16. To prevent meningococcal infection, the MenQuadfi® (Sanofi Pasteur, Lyon, France) vaccine was administered, and prophylactic ceftriaxone was administered for approximately 2 weeks. Platelet counts and lactate dehydrogenase concentrations gradually improved following treatment. However, renal dysfunction persisted. During the hospital course, the patient gradually developed fluid overload and cardiomegaly. On day 2, urine output decreased to 340 mL/day, corresponding to less than 0.3 mL/kg/h over 24 hours, consistent with KDIGO stage 3 acute kidney injury. In addition, persistent gastric discomfort raised concern for uremic symptoms; therefore, hemodialysis was initiated on day 3. The patient’s gastric discomfort resolved after starting dialysis, which suggested uremia as the underlying cause. To investigate the etiology of renal dysfunction, a renal biopsy was performed on day 7. A total of 18 glomeruli were obtained, of which 2 showed global sclerosis, and no segmental sclerosis was observed. Light microscopy showed thrombi within glomerular capillaries and double contours of the glomerular basement membrane (Fig. 1), which were consistent with TMA. Only mild to moderate interstitial fibrosis was observed, with no evidence of advanced tubular atrophy or extensive chronic scarring. Overall, the degree of chronic pathological change was limited and was considered a favorable prognostic feature. Immunofluorescence showed no notable immune complex deposition, while electron microscopy demonstrated marked subendothelial widening and a newly formed basement membrane without immune deposits (Fig. 1), further supporting the diagnosis of TMA. Treatment with ravulizumab and antihypertensive agents led to a progressive improvement in renal function, allowing discontinuation of dialysis on day 14. Sacubitril/valsartan was initiated on day 23 because of concentric arteriolar wall thickening resembling onion skin lesions observed in the biopsy specimen. His Cr concentration decreased from 3.79 mg/dL on day 22 to 2.0 mg/dL by day 29, accompanied by improved blood pressure control, and he was discharged home on day 32 (Fig. 2).

Anti-factor H antibody obtained before initiating ravulizumab was negative. Targeted resequencing of genomic DNA extracted from peripheral blood mononuclear cells using next-generation sequencing identified a heterozygous C3 c0.493G > T (p.Val165Phe, V165F) mutation. Although classified as a variant of unknown significance, this mutation has been reported in patients treated with eculizumab [19, 20], supporting the diagnosis of aHUS associated with *C3* gene mutation. With ongoing outpatient administration of ravulizumab every 8 weeks and management of chronic kidney disease, the patient’s renal function improved, with a Cr concentration of 1.23 mg/dL and urine protein-to-creatinine ratio of 0.17 g/gCr.

## Discussion and conclusions

We report a case of aHUS presenting with the triad of TMA and showing classic biopsy findings without immune deposits, in which a heterozygous C3 p.V165F mutation was identified. This missense variant in the macroglobulin 2 domain in the *C3* gene has been identified in only two patients in cohort studies of aHUS in Western populations [19, 20]. In these cohort studies, the presence of the C3 p.V165F variant was reported; however, detailed information regarding zygosity was not provided. Furthermore, the variant-specific clinical phenotype or detailed information regarding the response to eculizumab was not described in these two reports. Although pathogenic variants of C3 account for <10% of cases in Western populations, their prevalence is considerably higher in Japan, reaching approximately 31% [21]. However, no cases of the V165F mutation have been reported in Japan. Although C3 c.493G > T has been currently classified as a variant of unknown significance [20], the clinical course in the present case strongly suggests that this variant is more likely to be pathogenic. Val165 of C3 is located in the macroglobulin 2 domain, which is critical for interactions with the complement control protein domain of complement regulatory proteins, including complement factor H and membrane cofactor protein [22]. Although the precise mechanism remains undetermined, the V165F substitution may reduce the ability of C3b to interact with these regulators after its formation. This reduction in interaction may impair C3b degradation and predispose to excessive complement activation, ultimately leading to the development of aHUS.

The development of TMA in aHUS involves congenital factors and triggering events, such as infections and pregnancy. In the present case, approximately 2 months before the onset of aHUS, household coronavirus disease 2019 infection occurred among his family members, and the patient showed similar upper respiratory symptoms. SARS-CoV-2 can activate the alternative complement pathway and cause endothelial injury [23, 24]. Although the interval since the presumed infection was longer than usual, infection of this virus might still have acted as a triggering factor in the present case; however, this is difficult to confirm because the patient was not tested.

Our patient showed severe renal impairment at admission, with a serum Cr concentration of 10.2 mg/dL and an estimated glomerular filtration rate of 5 mL/min/1.73 m^2^, and he required temporary dialysis from day 3. Ravulizumab was initiated on day 2, and renal function subsequently improved to a Cr concentration of 3.79 mg/dL and an estimated glomerular filtration rate of 14 mL/min/1.73 m^2^ by day 22. In a clinical trial, ravulizumab provided rapid and sustained inhibition of C5, leading to hematological and renal improvement in adult patients with aHUS [25]. In this clinical trial, complete TMA remission at 26 weeks was achieved in 53.6% of patients. Additionally, a ≥25% improvement in Cr concentrations was observed in 58.9% of patients, with a baseline Cr concentration of 3.2 mg/dL and an estimated glomerular filtration rate of 10 mL/min/1.73 m^2^, indicating a milder degree of renal dysfunction compared with the present case. Early initiation of anti-C5 antibody therapy is associated with improved renal outcomes [26]. Therefore, the favorable outcome in the present case may have resulted from the early initiation of ravulizumab.

In aHUS, C5 inhibition is the standard of care. Although early initiation of C5 inhibition is crucial for renal recovery in aHUS, renal outcomes in cases complicated by HE are often suboptimal. In patients presenting with HE, renal survival appears less robust than in those without HE, suggesting complement-independent vascular injury superimposed on complement dysregulation in such cases [8]. Although hypertension is frequently observed in patients with aHUS and renin–angiotensin system inhibitors (RASis) are commonly administered [17], only a few case reports have described aHUS with HE/malignant hypertension managed with a C5 inhibitor plus an RASi (Table 2). In previously reported cases of malignant hypertension-associated aHUS (Table 2), most patients were treated with a combination of C5 inhibitors and RAS blockers, such as ACE inhibitors or ARBs. These combinations stabilized hematological parameters and provided partial improvement in renal function. However, serum Cr concentrations generally remained at approximately 2–3 mg/dL, or the studies did not specify renal outcomes in detail, indicating incomplete renal recovery [18, 27, 28]. Notably, in a report by Plasse et al. [29], renal recovery was not achieved despite dual RAS inhibition with telmisartan and aliskiren combined with eculizumab. This finding contrasts with our previous report, which suggested that early RAS inhibition provides renal benefits during the acute phase of malignant hypertension/HE-associated TMA [30]. Therefore, while RAS inhibition appears to play an important supportive role in aHUS with HE, conventional RAS blockade alone—or even dual RAS inhibition—may still result in only partial renal improvement when used along with complement inhibition.

**Table 2:** Adult cases of hypertensive emergency-associated atypical hemolytic uremic syndrome treated with C5 inhibitors and RAS blockade

First author	Year	Age (y)	Sex	BP (mmHg)	Cr at presentation (mg/dL)	Cr after treatment (mg/dL)	Onion skin lesion	C5 inhibitor	RAS blockade (ACEi/ARB/DRI/ARNI)	Genetic testing
Chen FY	2018	56	F	266/162	8.9	2.69	N/A	Eculizumab	Azilsartan	N/A
Plasse RA	2020	21	M	250/140	N/A (on dialysis)	N/A (on dialysis)	N/A	Eculizumab	Telmisartan, Aliskiren	−
Jiménez-Mayor E	2025	47	M	245/149	4.08	1.9	+	Eculizumab	Enalapril	−
Flindris K	2025	27	M	235/156	8.49	N/A	N/A	Ravulizumab	Irbesartan	CFH
**Present case**	**2025**	**57**	**M**	**198/102**	**10.19**	**1.23**	**+**	**Ravulizumab**	**Sacubitril/valsartan**	**C3**

F: female, M: male, BP: blood pressure, Cr: creatinine, RAS: renin–angiotensin system, ACEi: angiotensin-converting enzyme inhibitor, ARB: angiotensin receptor blocker, DRI: direct renin inhibitor, ARNI: angiotensin receptor–neprilysin inhibitor, CFH: complement factor H, C3: complement component 3, N/A: not available

In the present case, HE was observed at admission, and intravenous antihypertensive therapy with nicardipine was initiated. Although early initiation of ARNI in the acute phase of HE–associated TMA has been reported to be associated with potential renal benefit [14], this approach was intentionally deferred in the present case because severe acute kidney injury was present and aHUS was strongly suspected at admission. Initial treatment therefore prioritized suppression of complement activation with early C5 inhibition and blood pressure control using a calcium channel blocker. On day 23, an ARNI was introduced after renal biopsy revealed arteriolar wall thickening and thrombi within glomerular capillaries in only a few glomeruli, suggesting that the thrombotic tendency had largely improved with the initial treatment, whereas hypertensive vascular injury remained the predominant pathological feature. This intervention was followed by improved blood pressure control and further recovery of renal function (from 3.79 mg/dL on day 22 to 1.2 mg/dL 1 year later). This clinical sequence suggests that initiation of ARNI therapy after improvement of thrombotic activity, rather than during the acute thrombotic phase, may represent a reasonable therapeutic approach in aHUS cases complicated by HE and severe acute kidney injury.

Although HE is a common manifestation of complement dysregulation–associated TMA, to the best of our knowledge, this is the first reported case of aHUS complicated by HE treated with a combination of a C5 inhibitor and sacubitril/valsartan. In TMA, the glomerular filtration rate often declines in the acute phase because intraglomerular thrombi and arteriolar narrowing reduce renal perfusion. Arteriolar wall thickening reflects hypertensive microvascular remodeling, a pathological substrate in which the hemodynamic and endothelial effects of ARNI therapy may be particularly relevant. Unlike conventional RASis, ARNIs do not decrease glomerular perfusion pressure and may thus preserve renal blood flow while simultaneously exerting RAS inhibition and enhancing the natriuretic peptide pathway. These combined effects can improve glomerular hemodynamics, attenuate endothelial dysfunction, and limit microvascular remodeling beyond conventional ACE inhibitor/ARB effects [10, 11]. In biopsy-proven malignant-hypertension/HE-associated TMA, sacubitril/valsartan is associated with faster recovery of the kidney and better dialysis-free survival than ACE inhibitors/ARBs [14]. Although its independent effect remains unclear in aHUS-HE patients, ARNIs may represent a potentially effective adjunctive treatment option.

In the present case, the patient was of working age, and the greater convenience of ravulizumab was thus considered particularly beneficial. To date, there have been no clinical trials directly comparing ravulizumab and eculizumab. Ravulizumab is a long-acting anti-C5 monoclonal antibody with an extended half-life of approximately 50 days, allowing for a dosing interval of once every 8 weeks. A recent review suggested that both agents have similar safety and efficacy based on the results of their respective clinical trials. However, ravulizumab is often preferred because of its lower treatment burden, including reduced dosing frequency and potential economic advantages [31].

A recent systematic review suggests that gene mutations in C3, as well as in factor H and membrane cofactor protein, are associated with an increased risk of relapse [32]. Therefore, the use of ravulizumab may represent a more practical and effective treatment option in C3-aHUS, particularly when long-term C5 inhibition is required.

In conclusion, we report a case of C3 mutation-associated aHUS presenting with HE, in which sequential therapy with ravulizumab followed by sacubitril/valsartan resulted in sustained renal recovery. This case suggests that combining an ARNI with C5 inhibition may represent a promising strategy to improve kidney outcomes in aHUS complicated by HE.

## Data Availability

The datasets used in this case report are available from the corresponding author on reasonable request.

## Abbreviations

aHUS: Atypical hemolytic uremic syndrome
ACE: Angiotensin-converting enzyme
ARB: Angiotensin receptor blocker
ARNI: Angiotensin receptor–neprilysin inhibitor
BP: Blood pressure
C3: Complement component 3
C5: Complement component 5
CKD: Chronic kidney disease
Cr: Creatinine
eGFR: Estimated glomerular filtration rate
EM: Electron microscopy
HE: Hypertensive emergency
HPF: High-power field
IF: Immunofluorescence
LD: Lactate dehydrogenase
RASi: Renin–angiotensin system inhibitor
TMA: Thrombotic microangiopathy
UPCR: Urine protein-to-creatinine ratio
